# Effects of rainfall interception by sand-fixing vegetation on soil carbon and nitrogen distribution in a sand-covered hilly area

**DOI:** 10.3389/fpls.2025.1662481

**Published:** 2025-10-22

**Authors:** Wang Xin, Yang Zhenqi, Guo Jianying, Zhen Li, Qin Fucang

**Affiliations:** ^1^ Ecological Conservation and Restoration Laboratory, Ministry of Water Resources Pastoral Area Water Conservancy Science Research Institute, Hohhot, Inner Mongolia, China; ^2^ College of Desert Control Science and Engineering, Inner Mongolia Agricultural University, Hohhot, Inner Mongolia, China; ^3^ Inner Mongolia Autonomous Region Forestry and Grassland Seedling Station, Hohhot, Inner Mongolia, China; ^4^ Inner Mongolia Academy of Forestry Sciences, Hohhot, Inner Mongolia, China

**Keywords:** sand fixed vegetation, community structure, rainfall redistribution, soil organic carbon, total nitrogen

## Abstract

**Aims:**

The landscape of sand-covered hilly areas has been reshaped by afforestation in these areas. Dynamic changes in soil moisture and nutrients in forests after afforestation have become evident. However, clear studies have not focused on whether rainfall interception in these plantations affects soil concentration or concentration.

**Methods:**

This largely limits the development of effective management techniques for plantations and hinders the optimal utilization and management of water resources. In this study, an investigation was conducted on the plant community structure, rainfall interception characteristics, and soil organic carbon (SOC) and total nitrogen (N) concentrations or concentrations of three different plantations in the sand-covered hilly area of the Kuye River Basin. Grassland (Gl) was taken as the control.

**Results:**

The critical throughfall values for *C. korshinskii* (Ck), *S. Cheilophila* (Sc) and *P. sylvestris* (Ps) were 0.28, 1.78 and 2.04 mm, respectively. Corresponding stemflow critical values measured were 2.93, 1.08, and 3.30 mm, respectively. Ps exhibited the highest interception capacity, which was attributable to its dense canopy and layered branch architecture. Sc ranked second due to its larger leaf area, while Ck showed the lowest interception because of wide branch angles and smaller leaf area. Post-rainfall ground-level soil moisture and litter deposition are regulated by vegetation canopy structure in a direct way. SOC and N concentrations are subsequently controlled by these ground-level parameters. SOC concentration under Ps was 1.54 compared to that under Gl, while N concentration was 1.50 times higher, respectively.

**Conclusions:**

Thus, Ps demonstrates optimal effectiveness for improving soil quality in sandy hill restoration areas and merits continued implementation in this region.

## 1 Introduction

Sand-covered hilly areas are classified into arid and semi-arid regions. The precipitation level in such areas is low, and the frequency of extreme precipitation events has seen an increase owing to the impact of global climate change (Pei et al., 2023). Sand-covered hilly areas are the most typical and unique landscape in the dryland ecosystem. In these areas, sand-fixing vegetation plays a buffering role in breaking the wind, sand fixation and land degradation. Therefore, such vegetation is considered as an ecological barrier in this area (Liu and Du, 2022). In the past few decades, forest rehabilitation projects have been implemented as a part of slope agriculture in China, and numerous drought-tolerant trees and shrubs have been planted to reduce soil erosion (Bryan et al., 2018). These measures have significantly improved the vegetation coverage in this area and exerted a remarkable effect on soil and water conservation (Zhang et al., 2019a). Meanwhile, they have also resulted in changes to vegetation, soil properties and microbial community characteristics (Zhang et al., 2018). Nevertheless, the relationship between rainfall redistribution and soil organic carbon (SOC) and total nitrogen (N) during the construction of sand-fixing vegetation is unclear. Vegetation is an important factor affecting the SOC cycle. It participates in SOC cycling through root absorption and decomposition, as well as the reduction of litter and dead roots. Furthermore, different vegetation communities can form differing microtopographies, which causes differences in litter and decomposition rates (Tiessen et al., 1994). The community structure of plants exerts an influence on the rainfall interception process (Castro et al., 2006; Zhu et al., 2021). The change in soil nutrients is affected by vegetation (Li et al., 2021). To evaluate the effect of vegetation restoration and explore the resulting changes in soil composition, hence, it is necessary to comprehensively study and quantitatively characterize the process of rainfall interception (Lan et al., 2021).

The increased rainfall frequency with total unchanged rainfall amount increased SOC concentration, which mainly originated from increases in non-labile SOC concentration (Chen et al., 2020). The frequent occurrence of extreme rainfall events may greatly affect SOC fractions and carbon pool in the wet meadow of the Qinghai-Tibet Plateau (Wang et al., 2023). These studies have focused on the direct effects of rainfall on SOC, but the role of vegetation-mediated rainfall redistribution in regulating SOC and N remains underexplored. In forest ecosystems, the layers of canopy, herbaceous plants and litter intercept rainfall, divide it into interception, throughfall and stemflow, and influence soil moisture and nutrient dynamics (Gordon et al., 2020; de Queiroz et al., 2020). As a result, vegetation characteristics are key indicators for determining the amount of rainfall reaching soil (He et al., 2017; Wang et al., 2020). The characteristics of interception, stemflow, throughfall and litter interception have been described in extant studies (Ma et al., 2022). However, the effects of interception, stemflow, throughfall and litter interception of different sand-fixing vegetation types on SOC and N in forests need to be elucidated in more detail. In the process of artificial vegetation construction, the presence of different types of vegetation leads to differences in the composition of plant community and stand structure (Deng et al., 2018). Inevitably, this influences rainfall redistribution to varying degrees. The infiltration, storage and distribution of water in soil are deeply influenced by throughfall (Mei and Ma, 2022). This thus affects vegetation productivity and plant nutrient return (Yang et al., 2020), and furthermore soil nutrient characteristics (Liu et al., 2020). The effect of rainfall on SOC and N concentration characteristics is modulated by plant community and soil microbial decomposition (Fortier and Wright, 2021). Litter decomposition is a major factor that affects SOC and N concentration accumulation and cycling (Zhang et al., 2019b). The level of moisture entering different soil layers is different. Consequently, the nutrient concentration of each layer is different as well (Spyroglou et al., 2021; Simon et al., 2017). The effects of interception, stemflow, throughfall and litter interception on soil nutrient concentration and distribution after rainfall redistribution remain unclear despite their significance for elucidating the relationship between moisture and nutrient concentration during vegetation restoration.

Based on the above basic research, the association between rainfall redistribution and the dynamic changes in SOC and N for three kinds of sand-fixing vegetation in sand-covered hilly areas was examined in the present study. To address this gap, rainfall interception and the dynamic changes in SOC and N for three kinds of sand-fixing vegetation in sand-covered hilly areas were examined. The objective was to reveal the effects of rainfall redistribution on soil nutrient concentration, investigate the changes in nutrient levels under different sand-fixing vegetation types and clarify the coupling relationship between water and nutrients during vegetation restoration. The following research hypotheses were proposed: (1) Changes in vegetation structure lead to differences in the process of rainfall redistribution among sand-fixing vegetation types, and rainfall exerts a significant influence on throughfall, stemflow and canopy interception. (2) The cultivation of sand-fixing vegetation is beneficial to increasing SOC and N concentrations, with the most pronounced effects on the surface layer of soil. (3) The canopy characteristics of sand-fixing vegetation induce differences in the biomass and decomposition of the litter layer, which thereby substantially influences the concentrations of SOC and N in soil.

## 2 Materials and methods

### 2.1 Experimental site

**Figure 1:**
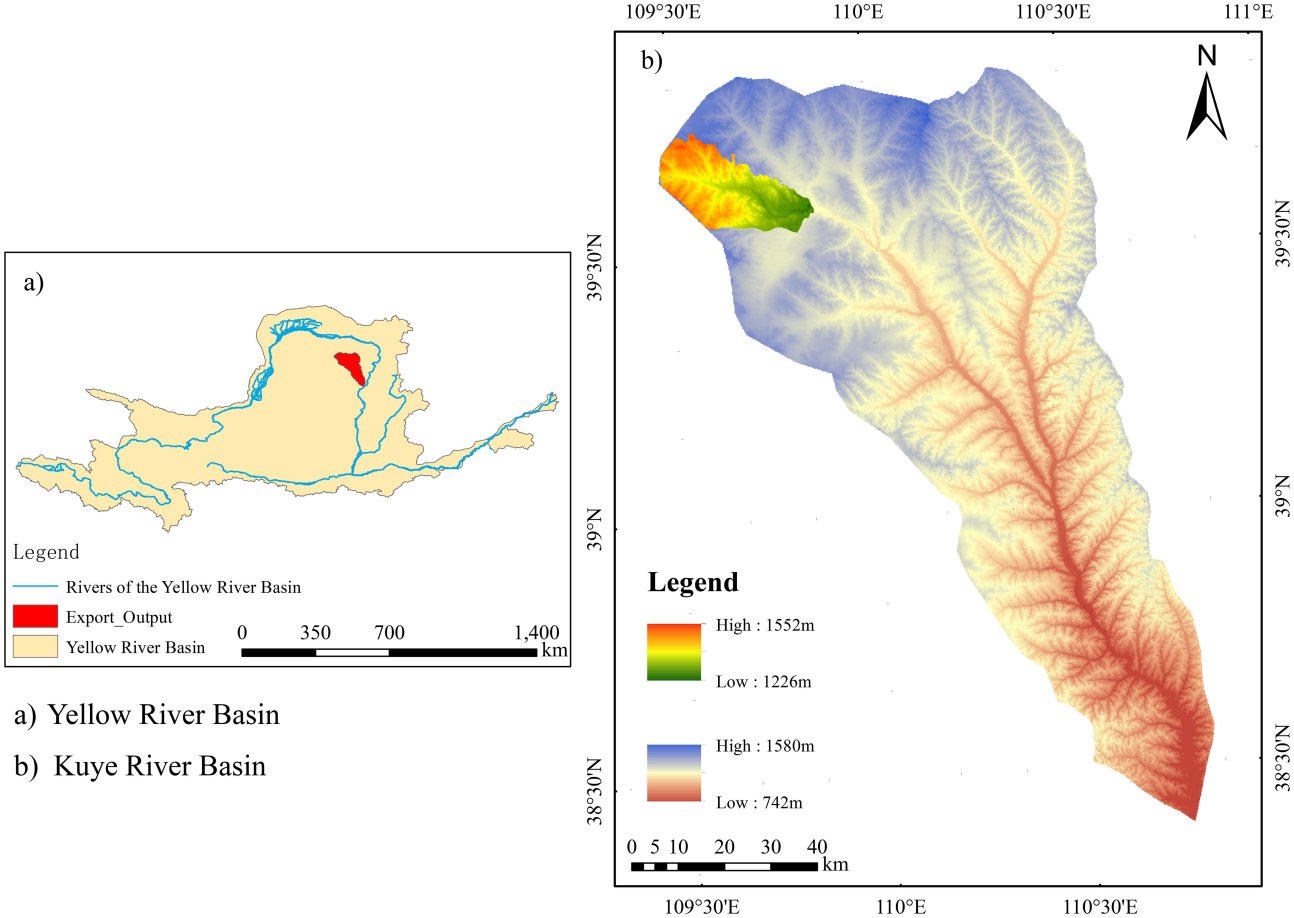
Map of the research region. **significant effects at p < 0.01 ,***significant effects at p < 0.001.

A typical artificial forest sample plot near the soil and water conservation monitoring station in the Inner Mongolia section of the Kuye River Basin was selected as the study area (**Figure 1**). It is located on the right bank of the upper reaches of the four-level tributary of the Yellow River Basin (109° 31′ 30.97′′ E, 39° 39′ 2.89′′ N). The landform types of the area encompass Pisha sandstone, chestnut soil, aeolian sandy soil, as well as sand-covered hilly and gully areas. The Miaochuan Basin located within the study area, has an arid and semi-arid temperate continental climate. The annual rainfall in the region averages 358.2 mm and ranges from 100.8 to 642.7 mm, with 2,900 sunshine hours annually. The effective accumulated temperature ≥10°C is 2,751.3°C and the mean annual evaporation is 2,563 mm. The prevailing wind in this area is northwesterly. Wind force varies from 5 to 8, with an annual wind speed of 3.6 m/s and a maximum instantaneous speed of 24 m/s.

### 2.2 Experimental design

The study area is situated in the Hetongchuanmiao section of the middle reaches of the Kuye River Basin. In June 2023, three common artificial sand-fixing vegetation types, namely Ck, Sc and Ps, were chosen as research objects. Sample plots of 20 m × 20 m and 15 m × 15 m were established for arboreal and shrub vegetations, respectively. Grassland (Gl) was used as a control. The characteristics of each stand were investigated and recorded (**Table 1**). Measurements were conducted for the average tree height, crown width, basal diameter, branch number, litter layer and biomass of trees and shrubs. The basal diameter of *C. korshinskii* (Ck) and *S. cheilophila* (Sc) is the total basal diameter obtained by adding the basal diameter of all branches on the ground (Luo et al., 2017). A field experiment was performed From July to October 2023. Nine standard trees were selected in each plot for the measurement of throughfall and stemflow.

**Table 1:** Characteristics of different types of vegetation in the study area.

Class	Vegetation	Height (m)	Average breast diameter (cm)	Average base diameter (cm)	Age (a)	CD (%)	HB (t·hm^-2^)	LB (t·hm^-2^)	LT (cm)
Shrub	*C.korshinskii*	2.9 ± 0.08b	–	20.7 ± 0.78	13	0.51 ± 0.03b	12.83 ± 0.53c	23.46 ± 1.61b	12.83 ± 0.02b
	*S. cheilophila*	3.02 ± 0.27b	–	28.66 ± 2.08	11	0.53 ± 0.03b	16.19 ± 0.61b	31.43 ± 3.74b	16.19 ± 0.57a
Arbor	*P. sylvestris*	4.17 ± 0.4a	38 ± 2.05	–	9	0.61 ± 0.01a	18.39 ± 1.18a	39.89 ± 3.80a	18.39 ± 0.12a

CD, Canopy density; HB, Herb biomass; LB, Litter biomass; LT, Litter thickness. Different lowercase letters in the same column indicate significant differences among different plantations, *p* < 0.05 level.

### 2.3 Investigation of vegetation characteristics

In June 2023, typical Ck, Sc and Ps forests near the contract temple soil and water conservation monitoring station in the contract temple basin of the Inner Mongolia section of the Kuye River Basin were selected for measurements. Later, sample sections of 20 m × 20 m and 15 m × 15 m were set up in each forest for the sampling of trees and shrubs, respectively. The space outside the field was used as a control. A measuring tape and a vernier caliper were utilized to measure the average height, diameter at breast height/basal diameter and biomass of trees and shrubs. Canopy density was determined on the basis of crown projection. The diameter at breast height of the tree refers to the diameter of the tree at a height of 1.1 meters from the ground. For Ck and Sc, the base diameter was used as the total base diameter obtained by adding the base diameter of all branches on the ground. The features of each stand were investigated and recorded (**Table 1**). The average value of each index was calculated. Standard plants were selected according to the average value. Nine standard plant vegetation types were chosen from each stand. Canopy density was the ratio of canopy projection area to forest area. Six 50 cm × 50 cm boxes were set up in each plot to collect litter. The thickness and weight of the litter layer were measured.

### 2.4 Investigation of soil characteristics

In the tree plot of 20 m × 20 m and the shrub plot of 15 m × 15 m, nine sampling points were selected. A soil sample from a depth of 0–150 cm was collected with a ring knife of 5 cm in diameter. Subsequently, soil samples from depths of 0-10, 10-20, 20-40, 40-60, 60-80, 80–100 and 100–150 cm were taken once. The collected soil was shade-dried, and its physical and chemical properties were determined. Soil samples were gathered in aluminum boxes to determine soil bulk density and soil mass water concentration (SMC, %). The method for determining SMC involved drying the sample in an oven at 105°C for 24 hours until it reached a constant weight. SOC (g·kg^-1^) was monitored by the dichromate titration-external heating method (Zhang et al., 2025). Soil N was determined by the Kjeldahl method (Cao et al., 2023).

The calculation formula for SOC density (SOCD) was as follows:


(1) SOCDi=SOCi×Bi×di×(1-Gi)

(2) $$\text{SOCD}=\sum_{\text{i}-1}^{\text{n}}\text{SOCi}\times \text{Bi}\times \text{di}\times \text{(1}-\text{Gi)}$$

The formula for the calculation of soil N density (SND) was as follows:


(3) SNDi=Ni×Bi×di×(1-Gi)

(4) $$\text{SND}=\sum_{\text{i}=1}^{\text{n}}\text{SNDi}=\text{Ni}\times \text{Bi}\times \text{di}\times (1-\text{Gi)}$$

In the formula, SOCDi represents the SOCD (kg·m^-2^) of a certain soil layer; SNDi stands for the SND of a certain soil layer (kg·m^-2^); SOCD denotes SOCD (kg·m^-2^); SND refers to SND (kg·m^-2^); SOCi means SOC concentration (g·kg^-1)^ in a certain soil layer; Ni is the N concentration of a soil layer (g·kg^-1^); Bi indicates the soil bulk density (g·cm^-3^) of a certain soil layer; di denotes the thickness of the soil layer (cm); Gi represents the percentage of the volume of gravel with a particle size greater than 2 mm. The soil particle size in this study is below 2 mm. As a result, Gi was ignored.

### 2.5 Observation of rainfall interception

Rainfall data were automatically collected by the long-term weather station. Rainwater was gathered in accordance with rainfall events, and six hours of rainfall were regarded as a rainfall event (Su et al., 2022). Rainfall was measured within 30 minutes after the end of the event (Zhang et al., 2021b). Ck, Sc and Ps plots were selected using a self-made rainfall collection device for forest throughfall observations (TF, mm). Nine standard plants were sampled from each plot. Within the projection areas of the selected standard plants, 12 self-made rain gauges were placed under each standard plant from the base to the four radiation directions. TF was calculated as follows:


(5) $$\text{TF}=\frac{1}{\text{n}}\sum_{\text{i}=1}^{\text{n}}\frac{\text{T}{\text{F}}_{\text{i}}}{{\text{A}}_{\text{i}}}$$

where TF represents throughfall, mm; TF_i_ stands for the volume of throughfall in the i^th^ throughfall collector, mm^3^; A_i_ refers to the rain area of the i^th^ throughfall collector, mm^2^; n means the number of throughfall collectors.

A polyethylene hose was spirally wound on the trunk. The hose was fixed with iron nails. The gap between the hose and trunk was sealed with glass glue. The stemflow of a single tree was converted into stemflow at the plot scale using the following formula (Tu et al., 2021):


(6) $$\text{SF}=\frac{\text{N}\times {\text{S}}_{a}}{\text{A}\times 10^{3}}$$

where SF represents stemflow, mm; N stands for the total tree in the sample plot; S_a_ refers to the mean stemflow of several standard trees, mL; A denotes plots (20 m × 20 m and 15 m × 15 m), m^2^.

The interception was calculated with the water balance formula as follows:


(7) Ic=P-TF-SF

where Ic represents interception, mm; P stands for rainfall, mm; TF is the amount of throughfall, mm; SF denotes stemflow, mm.

### 2.6 Statistical analysis

A normality test and a test for homogeneity of variance were carried out using IBM SPSS Statistics to ensure data validity. IBM SPSS Statistics 26.0 was applied to test the significance of the mean difference. Redundancy analysis was performed using CANOCO 5 software, followed by the implementation of partial least squares path modeling with the “plspm” package in R (v4.0.2).

## 3 Results and analysis

### 3.1 Rainfall redistribution characteristics of different forest stands

#### 3.1.1 Characteristics of throughfall

**Figure 2:**
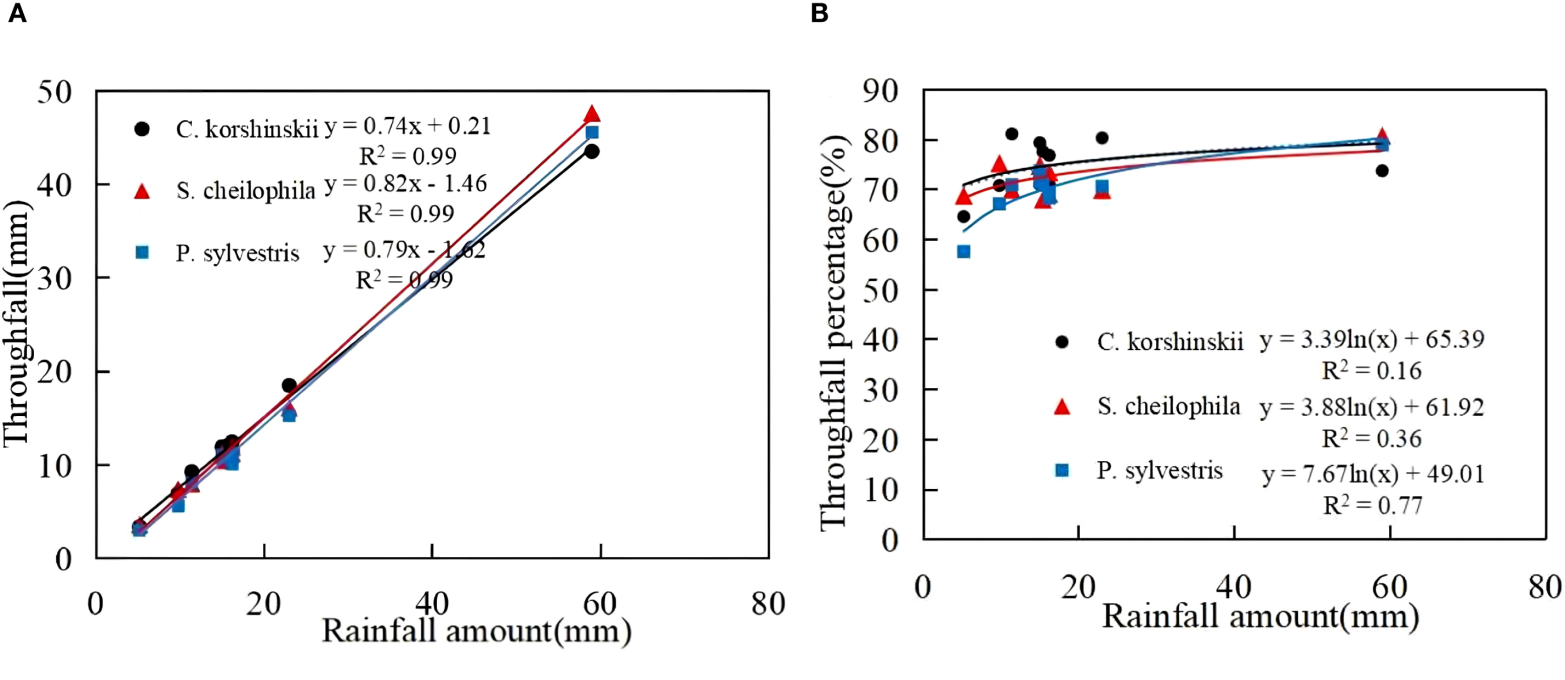
Characteristics of throughfall and its relationship with rainfall. **(A)** shows the throughfall characteristics of different sand-fixing vegetation types. **(B)** shows the throughfall percentage characteristics of different sand-fixing vegetation types.

In light of the rainfall redistribution data collected during the experiment in 2023, the total amount of throughfall for Ck, Sc and Ps was 140.02, 138.73 and 131.55 mm, respectively (**Table 2**). The total amount of throughfall (Equation 5) for each vegetation type accounted for 74.65%, 72.52%, and 69.96% of the total rainfall, respectively. A significant positive linear relationship was found between rainfall and throughfall for each vegetation type (R^2^ > 0.8, *p* < 0.01) (**Figure 2**). The throughfall for each vegetation type increased with the increase in rainfall. It can be seen from the equation that the threshold of throughfall produced by Ck, Sc and Ps were 0.28, 1.78 and 2.04 mm, respectively. A logarithmic relationship was detected between rainfall and throughfall rate for each vegetation type. When the rainfall was low, the throughfall rate increased rapidly, and the increase in throughfall rate gradually slowed down with the increase in rainfall.

**Table 2:** Statistical analysis of rainfall redistribution in different stands.

Vegetation	Throughfall			Stemflow			Interception			Rainfall (mm)
	Average (mm)	Total amount(mm)	Total throughfall rate	Average (mm)	Total amount(mm)	Total stemflow rate	Average (mm)	Total amount(mm)	Total interception rate	
*C.korshinskii*	14.00 ± 10.52a	140.02	74.65%	0.25 ± 0.12a	3.5	1.88%	4.27 ± 3.4a	42.68	22.92%	186.2
*S. cheilophila*	13.87 ± 11.69a	138.73	72.52%	0.29 ± 0.21a	2.91	1.56%	4.46 ± 2.4a	44.56	23.93%	
*P. sylvestris*	13.16 ± 11.26a	131.55	69.96%	0.28 ± 0.15a	3.8	2.04%	4.98 ± 2.7a	49.85	26.77%	

#### 3.1.2 Characteristics of stemflow

**Figure 3:**
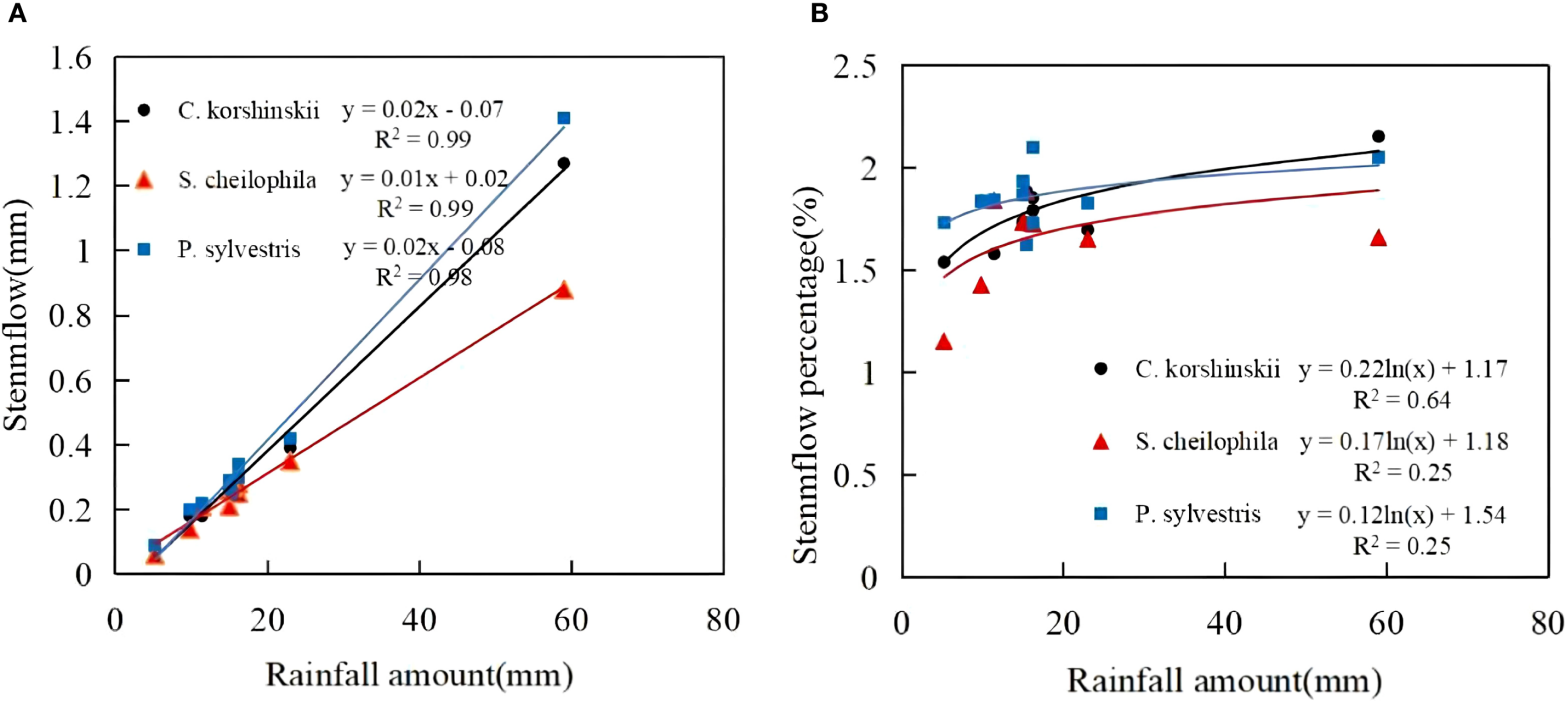
Characteristics of stemflow and its relationship with rainfall. **(A)** shows the stemflow characteristics of different sand-fixing vegetation types. **(B)** shows the stemflow percentage characteristics of different sand-fixing vegetation types.

During the experiment, the total stemflow of Ck, Sc and Ps was 3.5, 2.91 and 3.8 mm, respectively. The total stem runoff for each vegetation type occupied 1.88%, 1.56% and 2.04% of the total rainfall, respectively. A significant positive linear relationship was observed between the rainfall for each vegetation (Equation 6) type and stemflow (R^2^ > 0.9, *p* < 0.05) (**Figure 3**). The stemflow for each vegetation type showed an increasing trend with the increase in rainfall, as indicated by the equation. The threshold for Ck, Sc and Ps to produce stemflow was 2.93, 1.08 and 3.30 mm, respectively. A logarithmic relationship existed between rainfall and stemflow rate for each vegetation type. When the rainfall was low, the stemflow rate increased rapidly, and the increase of stemflow rate gradually slowed down with the increase in rainfall.

#### 3.1.3 Characteristics of interception

**Figure 4:**
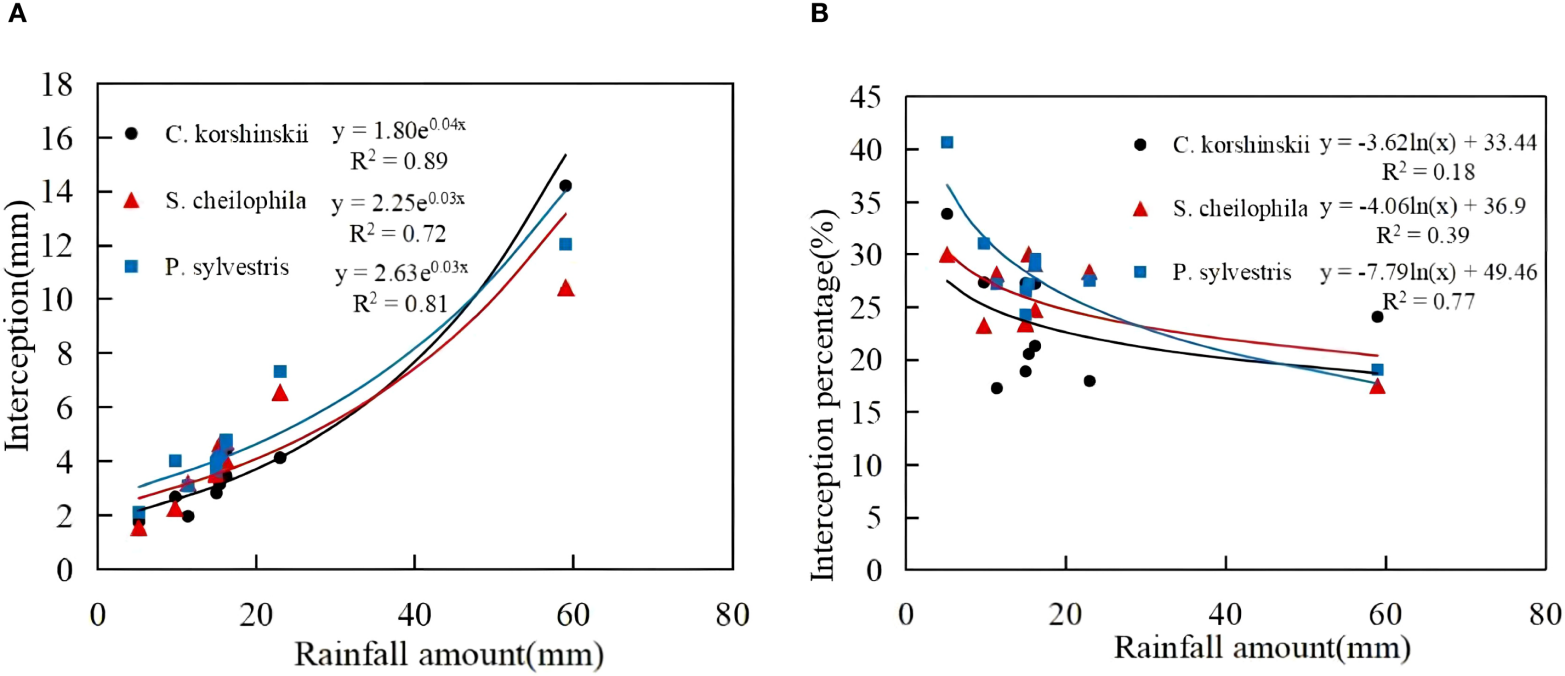
Characteristics of interception and its relationship with rainfall. **(A)** shows the canopy interception characteristics of different sand-fixing vegetation types. **(B)** shows the canopy interception percentage characteristics of different sand-fixing vegetation types.

During the experiment, the total interception of Ck, Sc and Ps was 42.68, 44.56 and 49.85 mm, respectively (Equation 7). The total interception for each vegetation type took up 22.92%, 23.93% and 26.77% of the total rainfall, respectively (**Figure 4**). A significant positive exponential relationship was noticed between the rainfall for each vegetation type and the total interception (R^2^ > 0.70, *p* < 0.05). The interception of each vegetation type rose with the increase in rainfall. A logarithmic relationship was noted between the rainfall and interception rate of each vegetation type. At the early stage of rainfall, the canopy was not saturated and the interception rate was high. With the increase in rainfall, a significant decrease occurred in the interception rate.

### 3.2 Changes in root biomass and soil bulk density in different vegetation types

**Figure 5:**
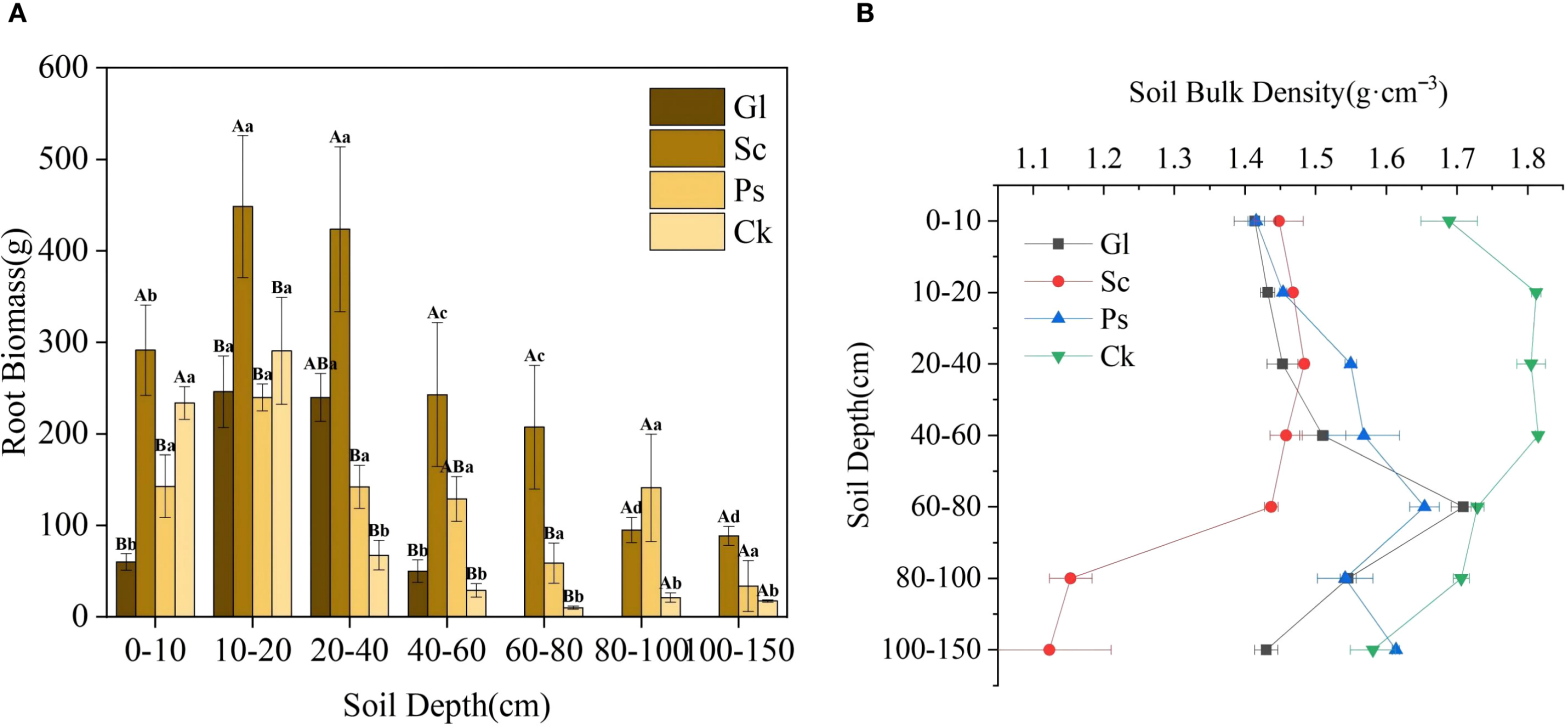
Root biomass and soil bulk density of each vegetation type. Gl, grassland; Sc, *S. cheilophila*; Ps, *P. sylvestris*; Ck, *C. korshinskii.* In **Figure 5A**, the capitalized letters stand for the significance of difference between different vegetation types on the same soil horizons, while small letters stand for the significance of difference between different soil horizons with the same plants. **(A)** shows the root biomass characteristics of different soil layers under different sand-fixing vegetation types. **(B)** shows the soil bulk density characteristics of different soil layers under different sand-fixing vegetation types.

Root biomass tends to rise before dropping with the increase of soil depth across different vegetation types. For all vegetation types, root biomass in the soil layer of 0–40 cm was significantly higher than that below 40 cm. The root biomass of Sc in the soil layer of 0–80 cm was greatly higher than that of other vegetation types. The soil bulk density of arbor and shrub forests, and Gl showed significant changes in different soil layers (**Figure 5**). In the 0–40 cm soil layer, the soil bulk density of Ck was higher than that of Sc, Ps and Gl, and ranged from 1.41 to 1.81 g·cm^-3^. In the 40–80 cm soil layer, the variation range of each vegetation type was 1.44 to 1.82 g·cm^-3^. The soil bulk density of Ck was the highest, and that for each vegetation type was not significant in the range of 40–60 cm. In the range of 80–150 cm, a remarkable change took place in soil bulk density for each vegetation type. The soil bulk density of Ck was higher than that of Sc, Ps and Gl, and the variation range was 1.12 to 1.71 g·cm^-3^.

### 3.3 Soil organic carbon and nitrogen distribution

#### 3.3.1 Distribution of soil organic carbon and total nitrogen

**Figure 6:**
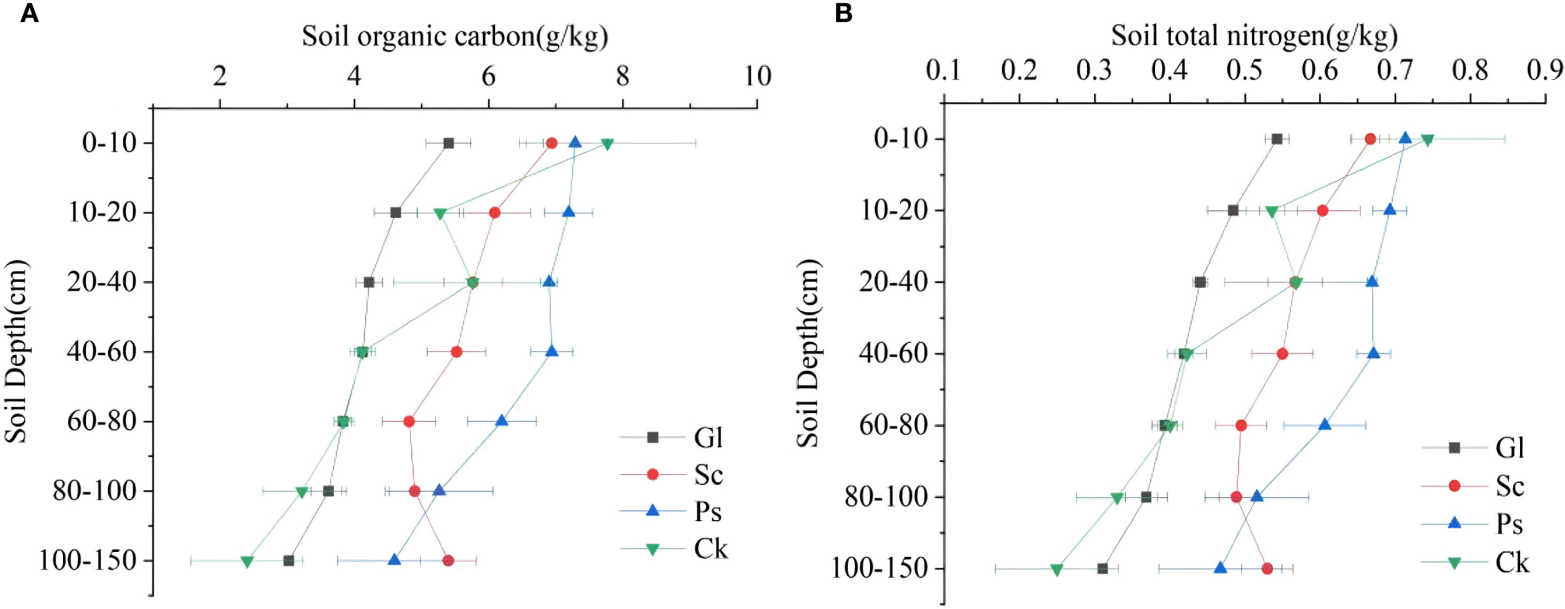
Distribution of soil organic carbon and total nitrogen in different vegetation types in different soil layers. **(A)** shows the soil organic carbon characteristics of different soil layers under different sand-fixing vegetation types. **(B)** shows the soil organic nitrogen characteristics of different soil layers under different sand-fixing vegetation types.

Two-way analysis of variance (ANOVA) showed that SOC and N were strongly affected by soil depth and vegetation type (**Table 3**). In general, the trend in the variation of SOC and N concentrations decreased with the increase in soil depth (**Figure 6**). The trend in the variation of SOC and N concentrations for each vegetation type was Ps > Sc > Ck > Gl. The SOC concentrations of Sc, Ps and Ck were 1.37, 1.54 and 1.23 times higher than that of Gl, respectively. The N concentration was 1.34, 1.50 and 1.04 times higher, respectively (*p* < 0.05). In the soil layer of 0–80 cm, the SOC concentration of Ps was significantly higher than that of Gl (*p* < 0.05). In the soil layer of 80–150 cm, the SOC concentrations of Sc and Ps were substantially higher than that of Gl (*p* < 0.05). Across all soil layers, the N concentrations of both Sc and Ps were largely higher than that of Gl (*p* < 0.05). In the soil layer of 0–40 cm, the SOC concentration of Ps was far above that of Gl. In the soil layer of 40–80 cm, the range of difference in SOC and N for Gl was not obvious, but a large fluctuation took place for Sc, Ps and Ck. The concentrations of SOC and N in Gl were significantly lower than those in sand-fixing vegetation.

**Table 3:** F values of two-way ANOVA.

Parameter	Soil depth	Vegetation	Soil depth×vegetation
SOC	22.64***	15.67***	7.25***
N	52.02***	15.72***	2.93***

****, significant effects at p<0.001.

#### 3.3.2 Distribution characteristics of soil organic carbon and nitrogen density in different vegetation types

**Figure 7:**
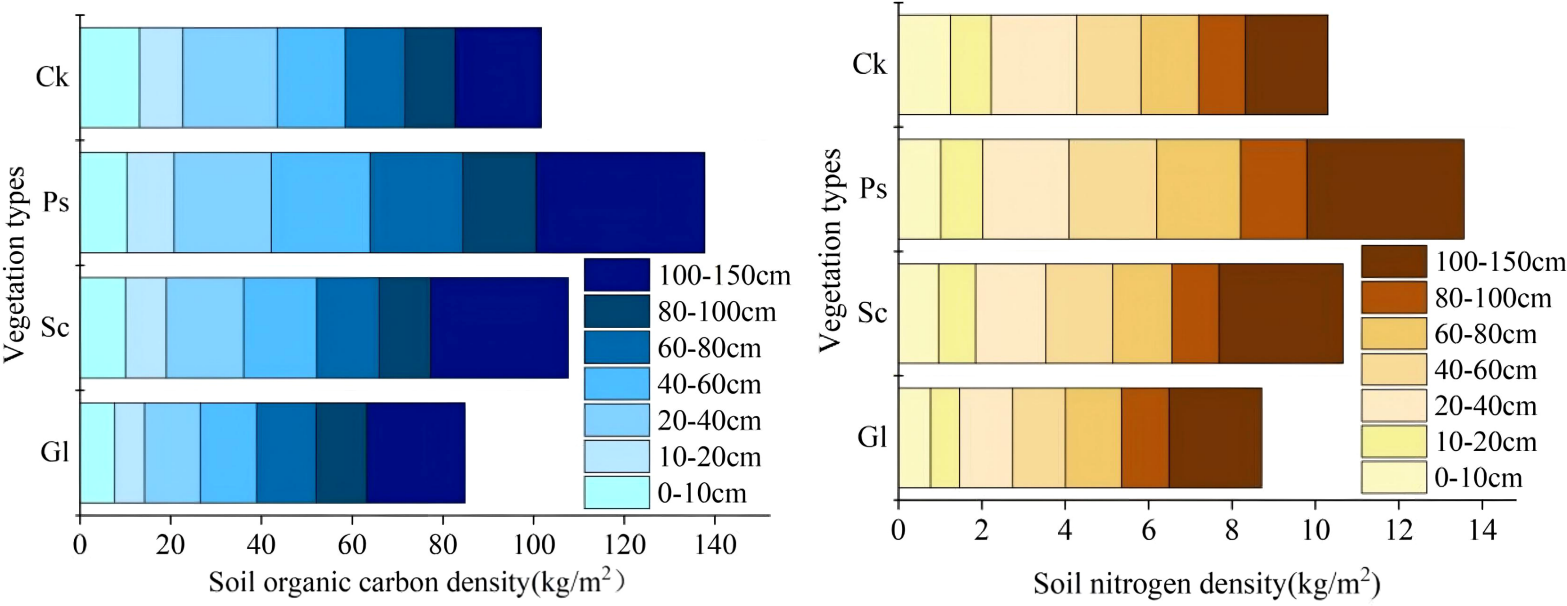
Carbon and nitrogen density distribution map of different vegetation types in different soil layers.

As shown in **Figure 7**, the SOCD (Equations 1, 2) of different vegetation types in the 0–150 cm soil layer fluctuated in the range of 137.76 to 101.73 kg·m^-2^. The SOCD and SND (Equations 3, 4) of Sc, Ps and Ck were significantly higher than that of Gl. The SOCD of Sc, Ps and Ck was 26.84%, 62.29% and 19.84% higher than that of Gl, respectively. The SND at a depth of 0–150 cm for different vegetation types fluctuated in the range of 13.57-10.30 kg·m^-2^. The SND of Sc, Ps and Ck was 22.41%, 55.72% and 18.22% higher than that of Gl, respectively. Compared with Gl, the establishment of sand-fixing vegetation significantly increased SOC and N density, especially in Ps.

### 3.4 Relationship between vegetation, soil and rainfall interception

**Figure 8:**
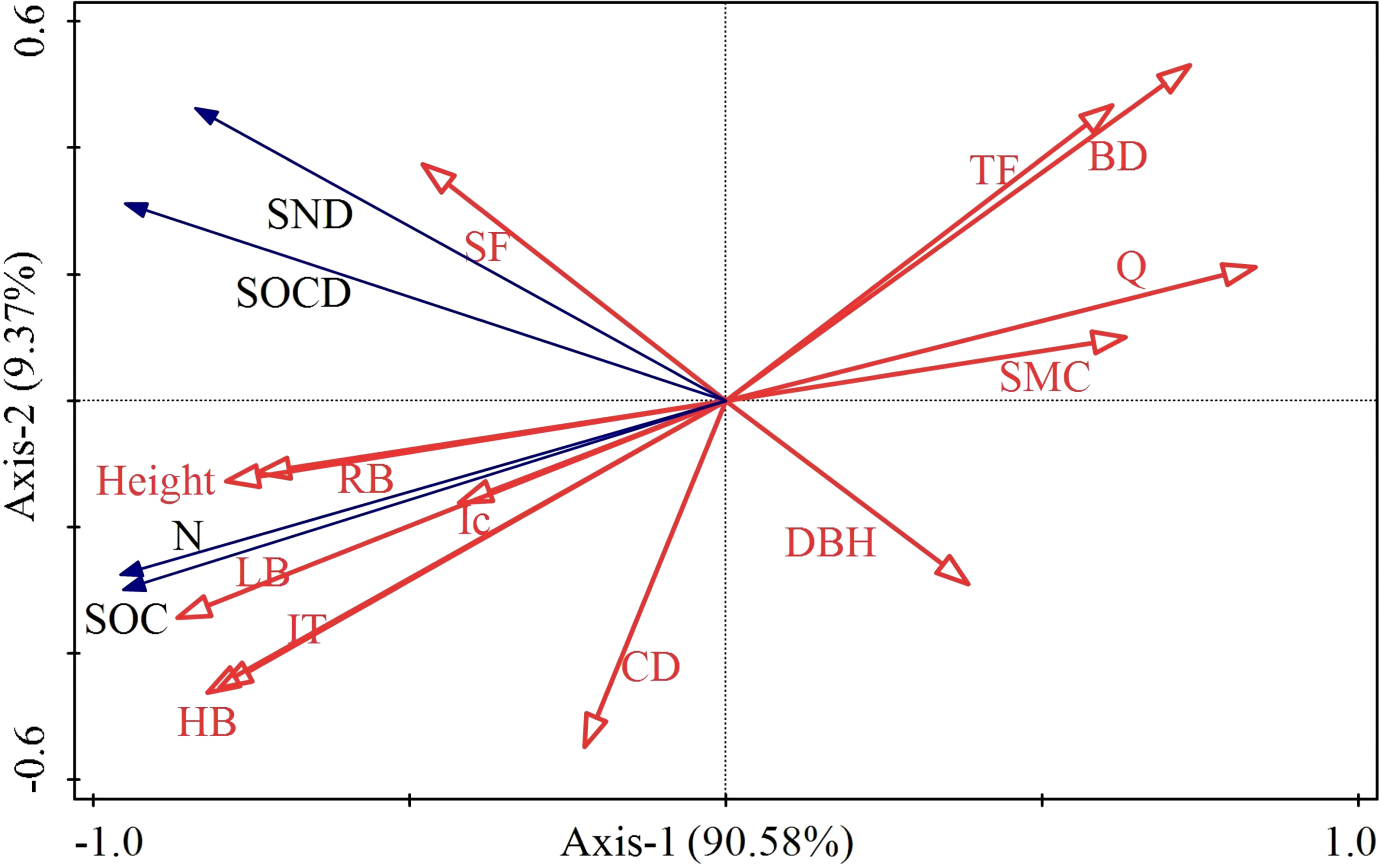
Redundancy analysis diagram. HB, Herbaceous biomass; LB, Litter biomass; LT, Litter thickness; CD, Canopy density; DBH, Diameter at breast height; Q, Vegetation Quantity; SMC, Soil mass water concentration; BD, Bulk Density; RB, Root biomass; Ic, Interception; SF, Stemflow; TF, Throughfall; SOC, Soil organic carbon; SOCD, Soil organic carbon density; SND, Soil nitrogen density; N, Soil total nitrogen.

**Figure 9:**
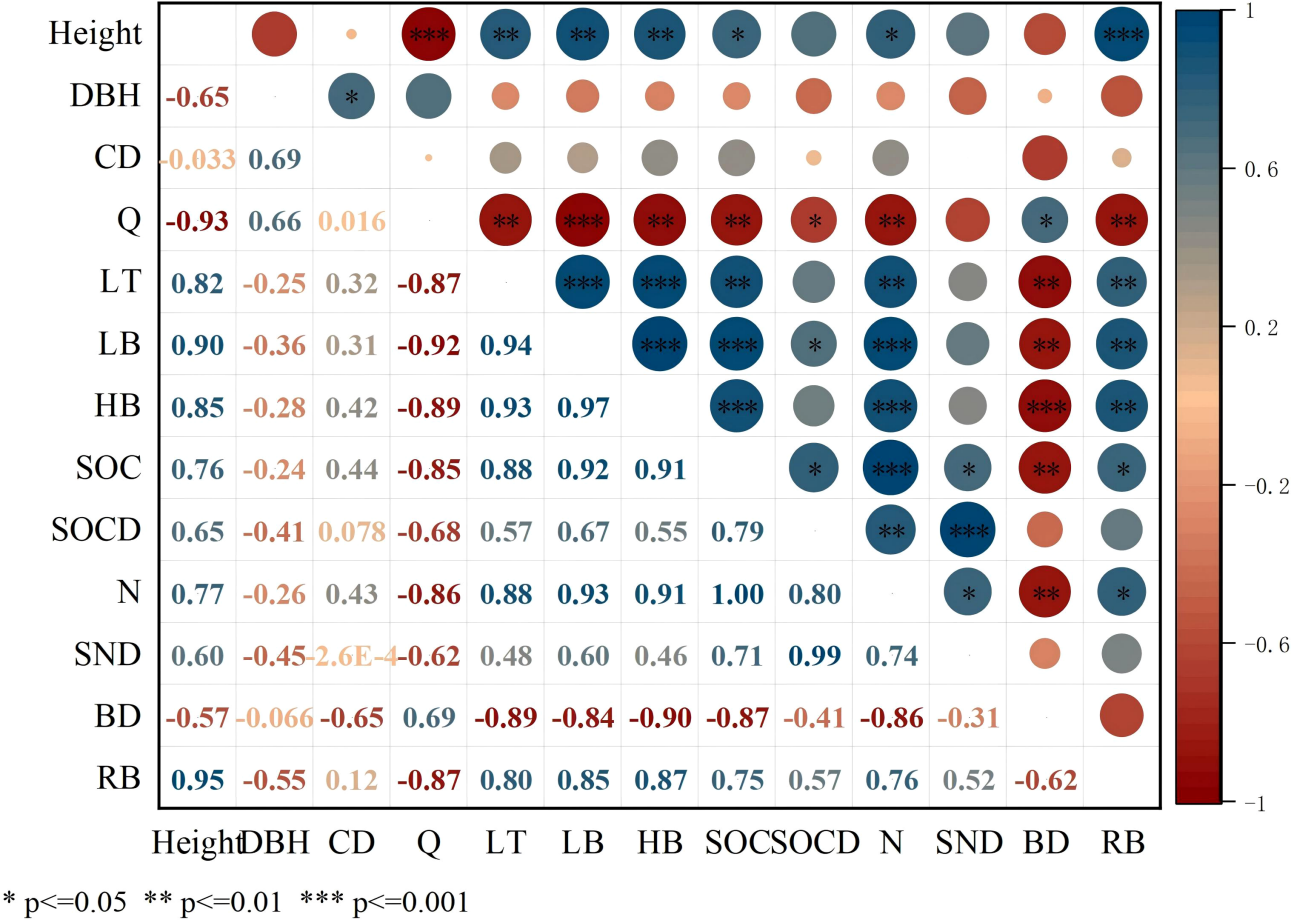
Pearson’s rank correlation coefficients. HB, Herbaceous biomass; LB, Litter biomass; LT, Litter thickness; CD, Canopy density; DBH, Diameter at breast height; Q, Vegetation Quantity; BD, Bulk Density; RB, Root Biomass; SOC, Soil organic carbon; SOCD, Soil organic carbon density; SND, Soil nitrogen density; N, Soil total nitrogen.

The redundancy analysis of vegetation and SOC and N revealed that the contribution rates of RDA1 and RDA2 were 90.58% and 9.37%, respectively (**Figure 8**). Evidently, height, RB, LT, LB, Ic and HB were correlated with SOC and N, while the correlation between SOCD and SND was relatively low. The RDA results showed that LB significantly influenced SOC, N, SOCD and SND (*p* < 0.01). Pearson’s correlation analysis indicated significant positive influences of height, LT, LB, HB and RB on SOC and N (*p* < 0.05) (**Figure 9**). Both Q and BD had a significant negative influence on SOC and N (*p*<0.05).

**Figure 10:**
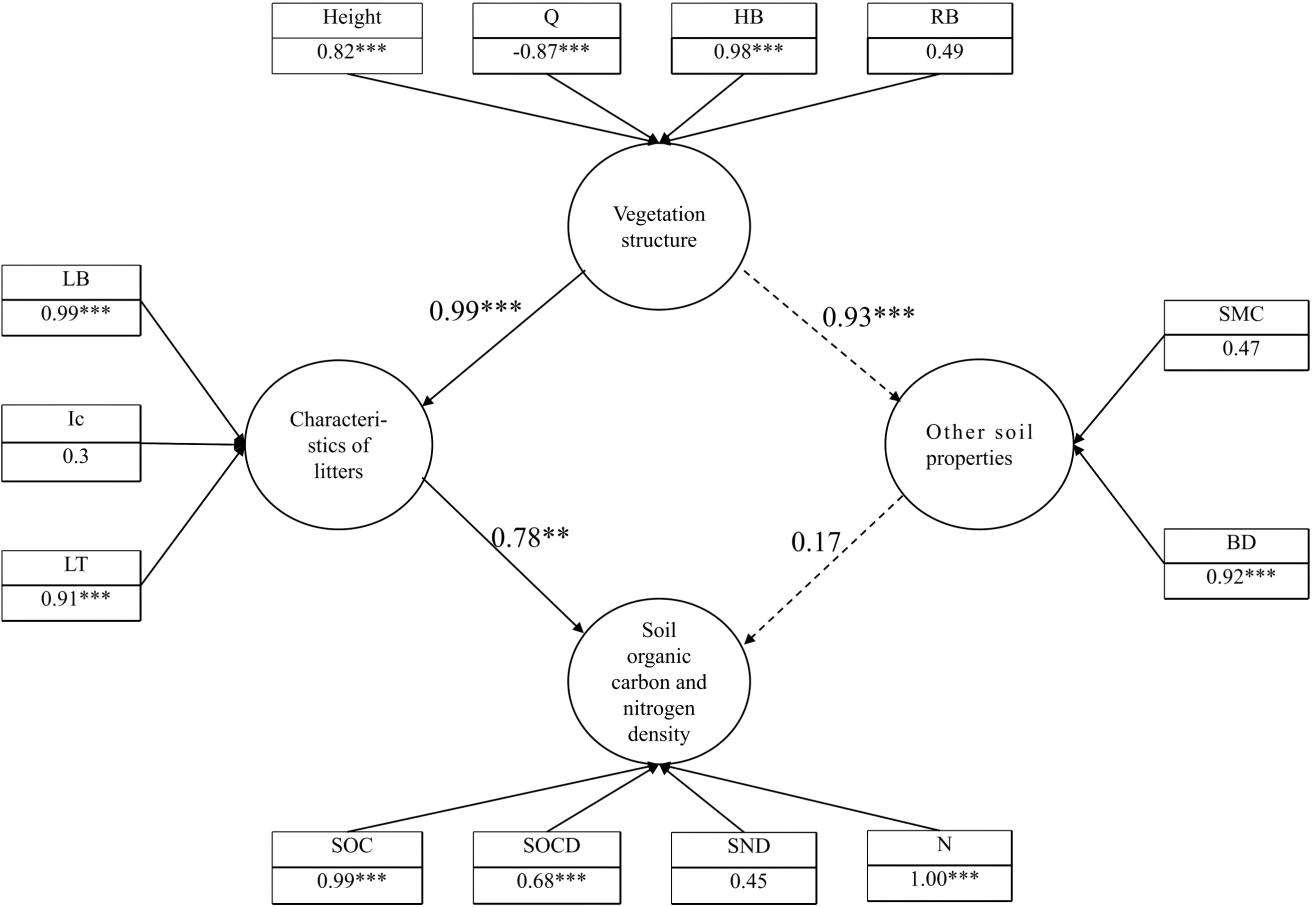
Structural equation model. **significant effects at p < 0.01 ,***significant effects at p < 0.001.

A structural equation model was developed, which demonstrated a good fit to the data (good of fitness (GoF) = 0.71). Based on the structural equation model, the direct or indirect effects of different sand-fixing vegetation types on SOC and N and their concentration or concentration through vegetation, interception and the litter layer, and SOC and N distribution were determined (**Figure 10**).

Finally, the results showed that the composition and structure of the litter layer were closely related to vegetation characteristics. LB was in turn a significant factor affecting SOC, SOCD, N and SND of vegetation areas (**Table 4**).

**Table 4:** F values of two-way ANOVA.

Parameter	Soil depth	Vegetation	Soil depth×vegetation
SOCD	403.92***	248.71***	7.96***
SND	345.26***	17.92***	2.93***

****, significant effects at *p*<0.001.

## 4 Discussion

### 4.1 Response of rainfall redistribution to different vegetation types

Due to the influence of canopy characteristics, different vegetation types use rainfall after rainfall in different ways. When rainfall was redistributed by the vegetation canopy, the largest proportion was throughfall, followed by interception and stemflow in succession (Zheng et al., 2020; Guo et al., 2023). In this study area, the throughfall rate of the shrub forest was higher than that of the arbor forest, and the stemflow and interception rates of the shrub forest were lower than those of the arbor forest. More than 65% of the rainfall in the three stands fell in the form of throughfall. The total interception occupied 22.92%-26.77% of the total rainfall (Levia and Herwitz, 2005). The connection between throughfall and rainfall may be affected by vegetation types and canopy morphological characteristics. Vegetation characteristics such as stand density, canopy density, branch roughness and branch length also have a bearing on rainfall redistribution (Liu et al., 2017). The throughfall and throughfall rate of *Ps* were substantially lower than those of other stands. Because the branches grew in layers and canopy density was high, the canopy had strong rainfall interception ability, which resulted in less throughfall (Guo et al., 2023). In this study, it was shown that the stemflow and stemflow rate of the Ps forest were the highest at 1.85%. This conclusion was basically consistent with the findings of Fan et al. (2019) that the stemflow of the Ps forest accounted for 2.54% of rainfall. This study found that Ps had the highest interception capacity, which was ascribed to its layered branch structure and high density. With its larger leaf area, Sc ranked second. Ck had the lowest interception on account of its wide branch angles and smaller leaf area. In summary, rainfall redistribution characteristics vary considerably among different forest types and are primarily influenced by leaf area, branch angle, branch roughness, crown width and other vegetation characteristics.

### 4.2 Response of soil characteristics to different vegetation types

#### 4.2.1 Response of root biomass and soil bulk density to different vegetation types

Soil bulk density, a basic physical property of soil, is affected by soil parent material, climate and biological disturbances, which significantly affects soil nutrients (Yu et al., 2019). Soil bulk density varied with soil depth across different artificial sand-fixing vegetation plots. It was found that bulk density increased gradually with increasing depth in Gl and Ps, whereas it increased initially and then decreased with depth in Sc and Ck. This variation was likely put down to the considerable differences in vegetation root distribution among these sand-fixing vegetation types (Wang et al., 2008). In the current study, soil bulk density was lower in the surface layer (0–20 cm), which was likely due to the higher density of plant roots in this zone (Wu et al., 2024). The root characterized by high plasticity can perceive and adapt to complex soil environmental factors (Zhang et al., 2021a). For example, roots were primarily distributed within the 0–40 cm soil layer in the Gl plot (Xiao et al., 2023), which resulted in the lowest bulk density at these depths. In contrast, Sc, Ck and Ps plots exhibited a greater concentration of primary roots in the upper soil layer. Land use types can have a significant influence on soil bulk density. In particular, shrubs have been shown to effectively reduce bulk density (Han et al., 2010). This is a finding aligned with the results of this study. Therefore, shrubs can be effective in improving the soil texture of sandy land (Han et al., 2010). Among the three artificial sand-fixing vegetation types studied, Sc demonstrated the lowest soil bulk density. This is primarily attributable to its extensive root system and dense branching structure, which promote litter accumulation. The subsequent microbial decomposition and synthesis of this litter generate substantial humus, and foster a loose, porous soil structure. This process reduces bulk density and consequently enhances the soil structure of sandy land.

#### 4.2.2 Response of soil organic carbon and nitrogen distribution to different vegetation types

ANOVA revealed that soil depth, vegetation type and their interaction (depth × vegetation) exerted significant effects on SOC and N concentrations. These findings are in line with those reported by Sun (2018). Vegetation is a major factor influencing the distribution and fixation of SOC. Chen et al. (1998) suggested that different types of plants have differential efficiency in forming plant residues and litter. Moreover, the spatial pattern of vegetation and the soil microenvironment created also differ. This will also affect the concentration and distribution of SOC (Liu, 2017), which thus affects SOC accumulation and distribution (Batzal et al., 2015). In this study, it was discovered that the concentrations of SOC and N in different soil layers of different sand-fixing vegetation forests were in the following order: Ps > Sc > Ck > Gl. Trees have complex litter, which can sustainably input SOC to soil. In the meantime, Gl litter is easy to decompose, and the input of SOC is not as good as that of trees, followed by shrubs. Thus, the SOC concentration of Gl was significantly lower than that of the other three vegetation types (Samuel et al., 2024). Chen (2020) pointed out that the standing biomass of Ps plantations is directly proportional to their carbon fixation capacity in northern China. In the present study, it was found that these plantations have remarkably higher biomass than other vegetation types and, consequently, the highest levels of SOC. The N concentration of tree and shrub soil was substantially higher than that of Gl soil. This may be because the presence of sand-fixing plant litter can effectively retain N in soil (Wang, 2017). The biomass of Gl was lower than that of the other three kinds of sand-fixing vegetation. Sand-fixing vegetation is rich in species, particularly litter species, and high in species diversity. As a result, the microbial biomass in soil is high and microbial activity is strong. This is conducive to decomposing organic matter on the soil surface (Singh et al., 2022). The vertical distribution of SOC is influenced by multiple factors, including litter input, soil leaching, microbial characteristics and plant root distribution (Zhang et al., 2023). Zhang (2023) stated that litter on the forest floor is the primary source of SOC. The contributions of litter and root systems to SOC vary with soil depth because of differences in SOC formation mechanisms (like leaching and microbial activities) and the vertical distribution of litter and roots. The results are in consistency with those of the present study. SOC concentration typically decreases with increasing soil depth. However, the specific vertical distribution patterns vary among different vegetation types (Wang et al., 2014). The mass fraction of carbon and N in the soil is therefore increased. Chen (2020) studied the Mu Us Sandy Land and noted that the carbon fixation capacity of 0-0.4 m soil was higher for Ps than for Gl. This was consistent with the conclusions of this study. In this study, SOC and N concentrations in different artificial sand-fixing vegetation plots decreased with the increase in soil depth. This indicates that SOC was positively affected by the accumulation and decomposition of litter in the surface layer of soil. This resulted in the obvious surface accumulation of SOC in different artificial sand-fixing vegetation plots, which was aligned with previous research findings (Wang et al., 2002). SOC is a vital factor affecting N level, and N concentration mainly depends on the accumulation of SOC (Gao et al., 2017). Therefore, the variation in N concentration for sand-fixing vegetation is basically in line with that of SOC concentration. The same results were obtained in this study.

### 4.3 Effects of rainfall interception by vegetation on soil organic carbon and nitrogen

Rainfall influences the concentration of active SOC in soil (Rasmussen et al., 1998; Kelleway et al., 2016; Franzluebbers et al., 2001). Litter is also a key factor of soil carbon sequestration (Liu et al., 2023). In addition, rainfall can change its chemical properties to a large extent (Ma et al., 2023). The concentration of compounds in litter, like lignin, tends to vary with rainfall, which can affect the degradability of SOC in soil (Yu et al., 2024). A drastic increase in rainfall influences the decomposition rate of litter to a great degree. Hence, the rainfall redistribution attributed to vegetation restoration can affect the distribution of carbon and N in soil (Victor et al, 2020). Wang et al. (2025) argued out that the increase in rainfall input can alleviate aridity and boost the growth and carbon input of plants. The present study showed that vegetation structure and rainfall redistribution directly influence litter. Litter is decomposed into organic and inorganic compounds—sources of SOC and N of soil (Zhang et al., 2018). LB promoted the increase of biomass in an indirect way (de Queiroz et al., 2020). The decomposition of plants increased N concentration, which thereby increased SOC, SOCD and SND concentrations. Wang (2023) studied Mediterranean ecosystems and noticed that long-term increased precipitation induced SOC loss via changes in microbial community composition, functional traits, root production and soil moisture. This study revealed that Ps exhibited the highest SOC concentration in spite of intercepting the most rainfall (resulting in the least reaching soil). This contrast demonstrates that SOC concentration is not solely determined by rainfall volume. Other critical factors include functional traits, root production, litter decomposition, microbial community composition and vegetation-specific redistribution patterns. Thus, future research should prioritize investigating these complex interactions. In this study, the impact of sand-fixing vegetation on soil nutrient concentration was explored by analyzing rainfall, canopy interception, and litter and soil layers. The findings offer valuable insights for vegetation restoration and plant species selection. The results also provide constructive advice on assessing the restoration of sand-fixing vegetation. Notably, the study highlights the significant role of litter in enhancing soil nutrients. Nevertheless, it does not include experiments on microbial activity or litter decomposition-key processes for the understanding of the mechanisms behind nutrient improvement. Thus, it is recommended that further research address these aspects.

## 5 Conclusions

This study elucidates how rainfall redistribution influences soil nutrient dynamics based on the distinct characteristics of different sand-fixing vegetation species. These findings provide critical guidance for selecting optimal vegetation species to restore ecosystems in sandy hills. Additionally, they clarify the essential coupling relationship between water redistribution and nutrient cycling during vegetation restoration.

1. Calculation shows that Ps, with the layered structure of its branches and large canopy cover, has the highest canopy interception. It is followed by Sc with broad leaves. Ck has the lowest canopy interception owing to its widely separated branches and small leaves.
2. The present study shows that the SOC and N concentrations in the understory soil of different sand-fixing vegetations drop with the increase of depth. In the depth between 0 and 150 cm, the concentrations of SOC and N in the soil of Gl, willow sand Ps and Ck decrease with the increase of depth. Various types of vegetation considered exhibit higher concentrations of SOC and N compared with Gl. Ps has the best performance in improving SOC and N in sand-covered hilly areas.
3. Redundancy and correlation analyses, and structural equation modeling indicate that the canopy structure of sand-fixing plants directly affects factors such as Ic, LB and LT. Furthermore, increases in LB and LT significantly enhance SOC, SOCD, N and SND.

## Data Availability

The raw data supporting the conclusions of this article will be made available by the authors, without undue reservation.

## References

[B1] Batzal N. Verrecchia P. E. Veterdal L. Lane S. N. (2015). Organic matter processing and soil evolution in a river system. Catena 126, 86–97. doi: 10.1016/j.catena.2014.10.013

[B2] Bryan B. A. Gao L. Ye Y. Sun X. Connor J. D. Crossman N. D.. (2018). China’s response to a national land-system sustainability emergency. Nature 559, 193–204. doi: 10.1038/s41586-018-0280-2, PMID: 29995865

[B3] Cao L. Xu M. P. Liu Y. S. Yu Z. C. Sun L. Tian X. F.. (2023). Response of soil nitrogen concentration or content and its vertical distribution to rainfall redistribution during Robinia pseudoacacia forest restoration on the Loess Plateau. Ecol. Indicators. 155, 111036. doi: 10.1016/j.ecolind.2023.111036

[B4] Castro G. Romero P. Gomez J. A. Fereres E. (2006). Rainfall redistribution beneath an olive orchard. Agric. Water Manage. 86, 249–258. doi: 10.1016/j.agwat.2006.05.011

[B5] Chen Z. Z. Wang Y. F. Tieszen T. (1998). Distribution of soil organic carbon in the major grasslands of xilinguole, inner Mongolia, China. Chin. J. Plant Ecology. 22, 545–551.

[B6] Chen W. Yang J. J. Yuan Y. Zhang H. Han F. P. (2020). Effects of artificial sand-fixing vegetation on soil nutrientsin Mu Us Sandy Land. Arid Zone Res. 37, 1447–1456. doi: 10.13866/j.azr.2020.06.09

[B7] Deng L. Wang K. B. Zhu G. Y. Liu Y. L. Chen L. Shang Z. P. (2018). Changes of soil carbon in five land use stages following 10 years of vegetation succession on the Loess Plateau, China. Catena 171, 185–192. doi: 10.1016/j.catena.2018.07.014

[B8] Fan G. H. Han C. Sun Y. T. Ge B. Zhuang J. Y. (2019). Rainfall redistribution in Pinus massoniana forest of Yangtze River Delta. Southwest China J. Agric. Sci. 32, 422–428. doi: 10.16213/j.cnki.scjas.2019.2.032

[B9] Fortier R. Wright S. J. (2021). Nutrient limitation of plant reproduction in a tropical moist forest. Ecology 102 (10), e03469. doi: 10.1002/ecy.3469, PMID: 34245567

[B10] Franzluebbers A. J. Haney R. L. Honeycutt C. W. Arshad M. A. Schomberg H. H. Hons F. M. (2001). Climatic influences on active fractions of soil organic matter. Soil Biology&Biochemistry 33, 1103–1111. doi: 10.1016/s0038-0717(01)00016-5

[B11] Gao Y. Peng D. Zhao Q. X. Liu J. L. Liu J. B. (2017). Effects of vegetation rehabilitation on soil organic and inorganic carbon stocks in the Mu Us Desert, Northwest China. Land Degradation Dev. 29, 1031–1040. doi: 10.1002/ldr.2832

[B12] Gordon D. A. R. Coenders-Gerrits M. Sellers B. A. Sadeghi S. M. M. Van Stan J. T. (2020). Rainfall interception and redistribution by a common North American understory and pasture forb, Eupatorium capillifolium (Lam. dogfennel). Hydrol. Earth Syst. Sci. 24, 4587–4599. doi: 10.5194/hess-24-4587-2020

[B13] Guo B. Yang H. Li J. C. Zhu C. Y. Zhao Y. H. Cao J. S.. (2023). Rainfall partitioning measurements for Pinus tabulaeformis forest in Taihang Mountains. Chin. J. Eco-Agriculture. 31 (12), 2011–2021. doi: 10.12357/cjea.20230172

[B14] Han F. P. Hu W. Zheng J. Du F. Zhang X. C. (2010). Estimating soil organic carbon storage and distribution in a catchment of Loess Plateau, China. Geoderma 154, 261–266. doi: 10.1016/j.geoderma.2009.10.011

[B15] He Z. Xiao P. Hao S. Yang C. (2017). Research progress on influence factors and models of rainfall redistribution under the action of vegetation. IOP Conf. Ser. Earth Environ. 69, 12034. doi: 10.1088/1755-1315/69/1/012034

[B16] Kelleway J. J. Saintilan N. Macreadie P. I. Skilbeck C. G. Zawadzki A. Ralph P. J. (2016). Seventy years of continuous encroachment substantially increase blue carbon capacity as mangroves replace intertidal salt marshes. Global Change Biol. 22, 1097–1109. doi: 10.1111/gcb.13158, PMID: 26670941

[B17] Lan Z. L. Zhao Y. Zhang J. G. Jiao R. Khan M. N. Sial T. A.. (2021). Long-term vegetation restoration increases deep soil carbon storage in the Northern Loess Plateau. Sci. Rep. 11, 13758. doi: 10.1038/s41598-021-93157-0, PMID: 34215791 PMC8253830

[B18] Levia D. F. Herwitz S. R. (2005). Interspecific variation of bark water storage capacity of three deciduous tree species in relation to stemflow yield and solute flux to forest soils. Catena 64, 117–137. doi: 10.1016/j.catena.2005.08.001

[B19] Li D. J. Wen L. Xiao K. C. Song T. Q. Wang K. L. (2021). Responses of soil gross nitrogen transformations to three vegetation restoration strategies in a subtropical karst region. Land Degrad. Dev. 32, 2520–2527. doi: 10.1002/ldr.3907

[B20] Liu X. D. (2017). Characteristics of soil labile organic carbon fractions in different communities of desert steppe (Ningxia University).

[B21] Liu X. Du H. (2022). Effects of different cropland reclamation periods on soil particle size and nutrients from the perspective of wind erosion in the mu us sandy land. Front. Environ. Sci. 10. doi: 10.3389/fenvs.2022.861273

[B22] Liu Z. B. Wang Y. H. Deng X. X. Liu Y. Zhang T. Zuo H. J.. (2017). Spatial variations of throughfall in a Larix principis-ruprechtii plantation of Liupan Mountains, Ningxia, China. Acta Ecologica Sinica. 37, 3471–3481. doi: 10.5846/stxb201602210305

[B23] Liu Y. Wang K. Dong L. Li J. Wang X. Shangguan Z.. (2023). Dynamics of litter decomposition rate and soil organic carbon sequestration following vegetation succession on the Loess Plateau, China. Catena 229, 107225. doi: 10.1016/j.catena.2023.107225

[B24] Liu Y. Zhu G. Hai X. Li J. Shangguan Z. P. Peng C. H.. (2020). Long-term forest succession improves plant diversity and soil quality but not significantly increase soil microbial diversity: Evidence from the Loess Plateau. Ecol. Eng. 142, 105631. doi: 10.1016/j.ecoleng.2019.105631

[B25] Luo Y. K. Fang J. Y. Hu H. F. (2017). Biomass estimation models and allocation patterns of 14 shrub species in Mountain Luya, Shanxi, China. Chin. J. Acta Phytoecological Sin. 41, 115–125. doi: CNKI:SUN:ZWSB.0.2017-01-013

[B26] Ma N. Ji Y. Yue K. Peng Y. Li C. Zhang H.. (2023). Effect of the seasonal precipitation regime on shrub litter decomposition in a subtropical forest. For. Ecol. Management. 548, 121423. doi: 10.1016/j.foreco.2023.121423

[B27] Ma C. K. Luo Y. Shao M. A. Jia X. X. (2022). Estimation and testing of linkages between forest structure and rainfall interception characteristics of a Robinia pseudoacacia plantation on China’s Loess Plateau. J. Forestry Res. 33, 529–542. doi: 10.1007/s11676-021-01324-w

[B28] Mei X. M. Ma L. (2022). Effect of afforestation on soil water dynamics and water uptake under different rainfall types on the Loess hillslope. Catena 213, 106216. doi: 10.1016/j.catena.2022.106216

[B29] Pei Y. Huang L. Shao M. Wang J. Zhang Y. (2023). Patterns and drivers of seasonal water sources of artificial sand-fixing plants in the northeastern Mu Us Sandy Land. Pedosphere. 34 (01), 63–77. doi: 10.1016/j.pedsph.2023.03.007

[B30] de Queiroz M. G. Silva T. G. Zolnier S. Souza C. A. Souza L. S. Araújo G. N.. (2020). Partitioning of rainfall in a seasonal dry tropical forest. Ecohydrol. Hydrobiol. 20, 230–242. doi: 10.1016/j.ecohyd.2020.02.001

[B31] Rasmussen P. E. Albrecht S. L. Smiley R. W. (1998). Soil C and N changes under tillage and cropping systems in semi-arid pacific northwest agrivulture. Soil&Tillage Res. 47, 197–205. doi: 10.1016/S0167-1987(98)00106-8

[B32] Samuel J. Liang G. Savannah A. Karen F. Jessica M. Bonnie W. (2024). Land use drives the distribution of free, physically protected, and chemically protected soil organic carbon storage at a global scale. Global Change Biol. 30, e17507. doi: 10.1111/gcb.17507, PMID: 39295217

[B33] Simon J. Dannenmann M. Pena R. Gessler A. Rennenberg H. (2017). Nitrogen nutrition of beech forests in a changing climate: importance of plant-soil-microbe water, carbon, and nitrogen interactions. Plant Soil. 418, 89–114. doi: 10.1007/s11104-017-3293-y

[B34] Singh G. Mishra D. Singh K. Shukla S. Choudhary G. R. (2022). Geographical settings and tree diversity influenced soil carbon storage in different forest types in Rajasthan, India. Catena 209, 105856. doi: 10.1016/j.catena.2021.105856

[B35] Spyroglou G. Fotelli M. Nanos N. Radoglou K. (2021). Assessing black locust biomass accumulation in restoration plantations. Forests 12, 1477. doi: 10.3390/f12111477

[B36] Su L. Yang J. Zhao X. Miao Y. (2022). Effects of fire on interception loss in a coniferous and broadleaved mixed forest. J. Hydrology. 613, 128425. doi: 10.1016/j.jhydrol.2022.128425

[B37] Sun L. (2018). The Dynamics of Soil Organic Carbon Fractions in Different Vegetation Types of Tianshan Forest (China: Xinjiang University).

[B38] Tiessen H. Cuevas E. Chacon P. (1994). The role of soil organic matter in sustaining soil fertility. Nature 371, 783–785. doi: 10.1038/371783a0

[B39] Tu L. Xiong W. Wang Y. Yu P. Liu Z. Han X.. (2021). Integrated effects of rainfall regime and canopy structure on interception loss: A comparative modelling analysis for an artificial larch forest. Ecohydrology 14, e2283. doi: 10.1002/eco.2283

[B40] Victor A. Valery N. Boris N. Aimé V. Louis Z. (2020). Carbon storage in cashew plantations in Central Africa: case of Cameroon. Carbon Management. 12, 25–35. doi: 10.1080/17583004.2020.1858682

[B41] Wang D. (2017). Relationship Between Soil Carbon and Plant Communities In TheYellow River Delta (China: LiaoCheng University).

[B42] Wang S. Huang M. Shao X. Mickler R. A. Li K. R. Ji J. J. (2014). Vertical distribution of soil organic carbon in China. Environ. Management. 33, 200–209. doi: 10.1007/s00267-003-9130-5

[B43] Wang Y. W. Liao C. Y. Sun C. Z. Xu H. (2008). Soil physical properties of sand-fixing forestsin Maowusu Sandland. J. Northwest Forestry University. 23, 36–39. doi: 10.19336/j.cnki.trtb.2009.04.013

[B44] Wang G. L. Liu G. B. Xu M. X. (2002). Effect of vegetation restoration on soil nutrient changes in Zhifanggou watershed of Loess Hilly Region. Bull. Soil Water Conserv. 22, 1–5. doi: 10.13961/j.cnki.stbctb.2002.01.001

[B45] Wang F. Pan X. B. Gerlein-Safdi C. Cao X. M. Wang S. Gu L. H.. (2020). Vegetation restoration in N orthern China: A contrasted picture. Land Degrad Land Degrad. Dev. 31, 669–676. doi: 10.1002/ldr.3314

[B46] Wang M. M. Sun X. Cao B. C. Chiariello N. R. Docherty K. M. Field C. B.. (2023). Long-term elevated precipitation induces grassland soil carbon loss via microbe-plant–soil interplay. Global Change Biol. 29, 5429–5444. doi: 10.1111/gcb.16811, PMID: 37317051

[B47] Wang M. M. Zhang S. Guo X. W. Wang G. C. Xia L. J. Xiao L. J.. (2025). Whole-profile soil carbon responses to concurrent warming and precipitation changes across global biomes. Global Change Biol. 31, e70105. doi: 10.1111/gcb.70105, PMID: 39995394

[B48] Wu Z. Xu C. Li R. Xu Y. Hua J. Sun S.. (2024). Full-field straw mulching and fertilizer application improved the soybean seed yield through optimization of the root and canopy structure: A study case in Huang-Huai-Hai region. Eur. J. Agronomy. 159, 127280. doi: 10.1016/j.eja.2024.127280

[B49] Xiao L. Leng M. Greenwood P. Zhao R. Xie Z. You Z.. (2023). Temporal and vertical dynamics of carbon accumulation potential under grazing-excluded grasslands in China: The role of soil bulk density. J. Environ. Management. 351, 119696. doi: 10.1016/j.jenvman.2023.119696, PMID: 38042080

[B50] Yang B. Zhang W. J. Meng X. J. Singh A. K. Zakari S. Song L.. (2020). Effects of a funnel-shaped canopy on rainfall redistribution and plant water acquisition in a banana (Musa spp.) plantation. Soil Tillage Res. 203, 104686. doi: 10.1016/j.still.2020.104686

[B51] Yu D. X. Jia X. X. Huang L. M. Shao M. A. Wang J. (2019). Spatial variation of soil bulk density in different soil layers in the Loess area and simulation. Acta Pedologica Sin. 56, 55–64. doi: 10.11766/trxb201802040086

[B52] Yu W. Wang C. Cornelissen J. Ye X. Yang X. Cui Q.. (2024). Precipitation and diameter affect wood decomposition both directly and indirectly via deadwood traits and position. Soil Biol. Biochem. 199, 109604. doi: 10.1016/j.soilbio.2024.109604

[B53] Zhang W. Gao D. X. Chen Z. X. Li H. Deng J. Qiao W. J.. (2018). Substrate quality and soil environmental conditions predict litter decomposition and drive soil nutrient dynamics following afforestation on the Loess Plateau of China. Geoderma 325, 152–161. doi: 10.1016/j.geoderma.2018.03.027

[B54] Zhang W. Liu W. C. Xu M. P. Deng J. Han X. H. Yang G. H.. (2019a). Response of forest growth to C:N: P stoichiometry in plants and soils during Robinia pseudoacacia afforestation on the Loess Plateau, China. Geoderma 337, 280–289. doi: 10.1016/j.geoderma.2018.09.042

[B55] Zhang F. Hou Y. Ao Y. Shen J. Jin K. (2021a). Root-soil interaction under soil compaction. J. Plant Nutr. Fertilizers. 27, 531–543. doi: 10.11674/zwyf.20318

[B56] Zhang Y. Tang Z. You Y. Guo X. Wu C. Liu S.. (2023). Differential effects of forest-floor litter and roots on soil organic carbon formation in a temperate oak forest. Soil Biol. Biochem. 180, 109017. doi: 10.1016/j.soilbio.2023.109017

[B57] Zhang J. Wang T. Wang J. Shen H. Li Y. Wu W. J.. (2025). Nutrient availability drives the ecological linkage between microbial functional diversity and soil organic matter molecular complexity during forest restoration. J. Environ. Management. 390, 126312. doi: 10.1016/j.jenvman.2025.126312, PMID: 40570419

[B58] Zhang W. Xu Y. D. Gao D. X. Wang X. Liu W. C. Deng J.. (2019b). Ecoenzymatic stoichiometry and nutrient dynamics along a revegetation chronosequence in the soils of abandoned land and Robinia pseudoacacia plantation on the Loess Plateau, China. Soil Biol. Biochem. 134, 1–14. doi: 10.1016/j.soilbio.2019.03.017

[B59] Zhang X. Zhao J. Lei L. Wang D. (2021b). Characteristics of rainfall redistribution of six shrubs in Eastern Qilian Mountain. Chin. J. Grassl 43 (01), 83–89. doi: 10.16742/j.zgcdxb.20190327

[B60] Zheng C. L. Jia L. (2020). Global canopy rainfall interception loss derived from satellite earth observations. Ecohydrology 13, e2186. doi: 10.1002/eco.2186

[B61] Zhu X. R. Liu H. Y. Li Y. Y. Liang B. Y. (2021). Quantifying the role of soil in local precipitation redistribution to vegetation growth. Ecol. Indicators. 124, 107355. doi: 10.1016/j.ecolind.2021.107355

